# The Impact of the COVID-19 Pandemic on the Epidemiology and Management Strategies of Forearm Fractures in Children—a Retrospective Study

**DOI:** 10.3390/children11121495

**Published:** 2024-12-08

**Authors:** Vlad Laurentiu David, Cristina Ana-Maria Garjoaba, Diana Popescu-Rohlicek, Larisa Anca Szentpeteri, Bogdan Ciornei, Calin Marius Popoiu, Eugen Sorin Boia

**Affiliations:** 1 Department of Pediatric Surgery and Orthopedics, “Victor Babes” University of Medicine and Pharmacy, 300041 Timisoara, Romania; david.vlad@umft.ro (V.L.D.); dianarohlicek@gmail.com (D.P.-R.); larisaflueratoru@gmail.com (L.A.S.); bogdan.ciornei@umft.ro (B.C.); mcpopiu@umft.ro (C.M.P.); boia.eugen@umft.ro (E.S.B.); 2 Department of Pediatric Surgery and Orthopedics, “Louis Turcanu” Emergency Children’s Hospital, 300011 Timisoara, Romania

**Keywords:** forearm fractures, COVID-19, pandemic, epidemiology, anatomopathology, management

## Abstract

Background: The COVID-19 pandemic has had a considerable influence over the management strategies in pediatric trauma all over the world. We are making a comparative assessment of all pediatric forearm fracture presentations in a tertiary center in Romania in a pre-pandemic year 2019 (NPG) versus a pandemic year 2021 (PG). Material and Methods: We retrospectively compared the epidemiological, the anatomopathological, and the management features of forearm fractures for the two years. Results: A total of 1403 patients with forearm fractures, 720 in NPG and 683 in PG, ages < 1 year–19 years (mean for NPG = 9.38 years and mean for PG = 9.39 years), were included in the study. There are no differences in demographics of the patients. There was an increase in the angulation ratio in the PG group and no other differences in the anatomopathological features. Most of the patients were treated by non-surgical means with no differences between groups. In PG, titan elastic nails were more often used than K-wire for the stabilization of diaphyseal fractures. There was no difference in terms of complication rates and reinterventions between groups. The number of hospital admissions (*p* < 0.01) and the length of hospital stay were significantly higher in NPG versus PG, (*p* < 0.01). Conclusions: The COVID-19 pandemic has had little impact on the epidemiology, anatomopathological features, and management strategies of forearm fractures in children. The only significant change in the medical strategy in our series was towards reducing the hospitalization rate and duration, reducing the follow-up visits.

## 1. Introduction

Forearm fractures are among the most common traumatic lesions in children and an important cause for emergency room presentations [1]. The incidence of forearm fractures in children range from 140/100,000 person/year to 317/100,000/year depending on the geographic area, and it has been estimated that approximately one-third of all children suffer at least one fracture before the age of 17 years [1,2]. These fractures can have a profound impact on a child’s physical functioning, general well-being, and overall quality of life. Forearm fractures in children were most common in children aged 5–14, with a higher incidence among boys due to increased participation in outdoor and sports activities. The primary causes include falls from a height, playground injuries, and sports activities such as cycling, skateboarding, and soccer [1,2].

Despite being common and often deemed benign lesions that generally heal well with proper treatment, improperly managed forearm fractures can result in significant complications. Depending on the type and severity of the fracture, there are various methods of management of forearm fractures in children, ranging from immobilization only to closed or open reduction and surgical stabilization [3,4]. Thus, the management of pediatric forearm fractures demands careful consideration to ensure optimal functional and aesthetic outcomes.

The outbreak of the COVID-19 pandemic in late 2019 has dramatically transformed the healthcare landscape, imposing unique challenges and disruptions across various medical specialties [5,6]. The pandemic has compelled healthcare systems worldwide to adapt swiftly, altering the delivery of care and influencing patient outcomes. For pediatric trauma, the tendency was to minimize the number and time of presentations to the emergency departments and the number and time of hospital admissions. The goal was to minimize physical contact with patients and reduce the burden over the health system and preserve the much needed material and human resources elsewhere [7,8,9,10,11]. These adaptations likely influenced the epidemiology of pediatric trauma, management approaches, and treatment outcomes, warranting an in-depth evaluation of their effects. The COVID-19 pandemic significantly altered the patterns of pediatric forearm fractures, primarily through changes in injury mechanisms, delays in care, and the adoption of telemedicine for initial consultations [12,13,14,15].

## 2. Materials and Methods

We performed a comparative assessment of all pediatric forearm fractures presentations in a tertiary center in Romania in a pre-pandemic year (2019) versus a pandemic year (2021). The study seeks to understand the effects of the COVID-19 pandemic on the epidemiology of forearms fractures in children, the change in management strategy and treatment outcomes, and to understand how these effects may influence our future approach for this type of lesion.

The study was performed in tertiary pediatric trauma center in Timisoara, Timis county, Romania, dedicated exclusively to children and being the sole center for pediatric trauma over a population of approximately 750,000 inhabitants. The study was performed in line with the principles of the Declaration of Helsinki and was approved by the Ethic Committee of the “Louis Turcanu” Emergency Children’s Hospital Timisoara, Romania No. 19766/20.12.2021. We retrospectively acquired all the records (electronic registry of the hospital, written registry of the emergency department and pediatric orthopedics department, patients’ charts) of patients with forearm fractures for the years 2019 and 2021, respectively. We recorded and assessed the following parameters: age, sex, anatomopathological characteristics of the forearm fractures (localization, closed/open fractures, involved bones, displaced/non-displaced, multiple fractures, polytrauma), admissions, treatment method and stabilization, initial X-ray assessment and follow-up, clinical follow-up, timing of cast and/or osteosynthesis material removement, complications, re-admissions and reintervention. These parameters were comparatively assessed between the two groups: non-pandemic group, year 2019 (NPG) and pandemic group, year 2021 (PG).

Fractures were classified according to multiple parameters, if one or both forearm bones were involved, the location of the fracture was described as epiphyseal (Salter–Harris fractures involving the epiphysis), distal fractures involving the distal metaphysis, diaphyseal, and proximal. Another differentiation was made between non-displaced, angulated, and displaced fracture, as well as the type of treatment the patients had received, either surgical or non-surgical.

Non-surgical approaches meant either simple casting with plaster of Paris or reduction under sedation with NO2 administered by means of a facial mask and subsequent casting. These techniques were used for simple, non-displaced or stable angulated fractures of one or both bones of the forearm.

The surgical techniques used distal and epiphyseal fractures included closed or open reduction and pinning with Kirschner wires, diaphyseal fractures benefited from elastic stable internal fixation by means of nailing with elastic nails made of titanium (TEN).

Statistical analysis was performed using IBM SPSS Statistics version 23. The data were retrospectively extracted from the records, the data were not calibrated in any way and no blind evaluation was performed. Descriptive data are reported as mean values and standard deviations, medians or percentages. The chi-square test was used to assess the relationship between non-numeric parameters and the independent sample (Student’s) t-test to compare the continuous numerical variables for the two groups (during and before the pandemic). The significance level (*p* value) was set at 0.05.

## 3. Results

A total of 1403 patients with forearm fractures, 720 in NPG and 683 in PG, age < 1 year–19 years (mean for NPG = 9.38 years and mean for PG = 9.39 years), were included in the study. Most of the patients were male 944 versus 459 female patients, with equal distributions between groups. There are no differences in demographics, age, and weight of the patients, most of the patients (73% in NPG, 71% in PG, *p* > 0.05) were living in the area comprising 50 km radius from the hospital. Displacement (lateral displacement or overriding of the fragments) or angulation of the fractured bones were found in 673 patients (48%). There was an increase in the angulation ratio in the PG group (*p* < 0.01). There were no other differences in the anatomopathological features of the fractures between groups. There were 19 open fractures (11 in NPG and 8 in PG). The anatomopathological characteristics of the fracture types are presented in Table 1.

**Figure 1:**
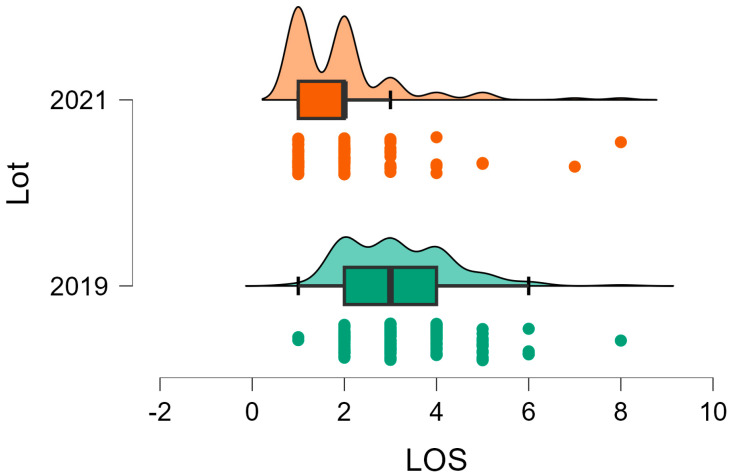
Length of stay NPG (2019) versus PG (2020).

A total of 403 patients were admitted in the hospital, 264 in NPG and 139 in PG (*p* = 0.00). Mean hospital stay was 3.07 days for NPG (1–9 days) and 1.88 days for PG (1–8 days), *p* = 0.00 (Figure 1).

Most of the patients, 1127, were treated by non-surgical means, 568 in NPG and 559 in PG (*p* > 0.05). Non-surgical closed reduction and arm casting of the fracture was performed in 341 cases (151 in NPG and 190 in PG, *p* > 0.05) and cast immobilization only in 792 patients. Surgical treatment was necessary in 275 patients (152 in NPG and 123 in PG, *p* > 0.05). The choice of surgical method of treatment was according to the type of fracture and preferences of the surgical team: distal fractures were treated by means of closed reduction and pining with K-wires (127 cases, 76 in NPG versus 51 in PG, *p* > 0,05) while in diaphyseal fractures, titanic elastic nails (TEN) were the main option (65 cases, 23 in PG and 43 in NPG). In one case of iterative radius diaphyseal fracture, stabilization was achieved by using plate and screw (Table 2). Secondary displacement occurred in 42 patients who were initially treated by orthopedic means (23 in PG and 19 in NPG, *p* > 0.05). Secondary intervention was necessary for 42 patients, closed reduction and cast in 12 patients and surgery in 30 patients. There were no other complications than the abovementioned cases of secondary displacement. Readmission was necessary in 35 patients (12 in NPG and 23 in PG), *p* = 0.04).

In relation to the treatment option for each type of fracture, we noticed one statistically significant difference (*p* = 0.03) in PG, TEN was more often used than K-wire for the stabilization of diaphyseal fractures. For all other types of fractures, the percentages were similar (Table 2).

**Figure 2:**
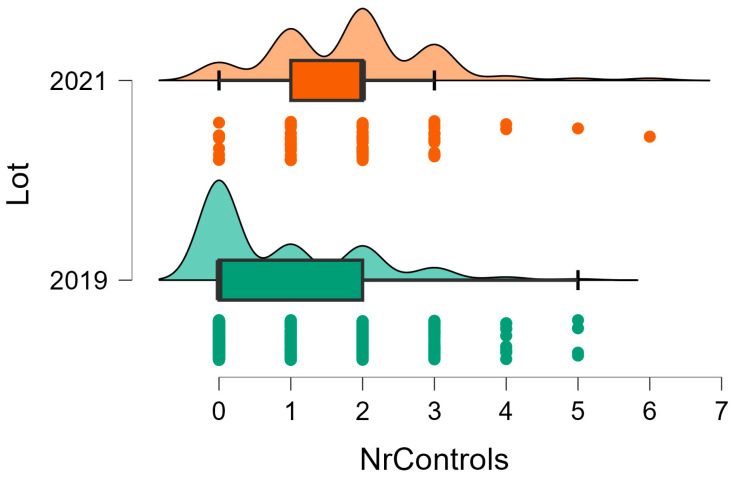
Number of patients followed up at our institution NPG (2019) versus PG (2021).

Follow up was conducted at our institution in 354 out of the 1403 patients treated by us for forearm fractures (25.2%). There was a significant difference concerning the follow up of the patients between NPG (279 patients, 38.7%) and PG (75 Patients, 10,9%, *p* < 0.01) (Figure 2). Mean visit/patient was similar with 1.85/patient in NPG versus 2.05/patient in PG (*p* > 0.05). Removal of the osteosynthesis material was performed on 210 patients (111 in NPG and 99 in PG, *p* > 0.05). Readmission for the removal of the osteosynthesis material was considered necessary in 103 of the 111 NPG patients (92.7%, mean 2.84 days/patient) versus 75 of the 99 patients in PG (75.7%, mean 1.47 days/patient, *p* = 0.00).

## 4. Discussion

The COVID-19 pandemic has significantly influenced various aspects of pediatric health, including the incidence and management of trauma in children. Our goal with this study was to seek the degree to which the social restrictions and changes in the healthcare strategies in our geographic area impacted the demographics, clinical characteristics, anatomopathological characteristics, and the way we treat forearm fractures in children. To this extent, one of the first noteworthy aspects that we have seen was that there were no changes in the incidence and demographics of forearm fractures during the pandemic year. We observed only a less than 5% decrease incidence for PG and no alteration in sex ratio or age pattern between the two groups. This came somehow like a surprise, considering the social distance policy, home schooling, and reduced social and sports activities imposed during the pandemic. This “paradoxical” results are consistent with those of similar studies. One study analyzed the US National Electronic Injury Surveillance System and observed that after an initial decrease in the incidence of pediatric fractures in 2020, the numbers of Emergency Department presentations rose back to the pre-pandemic years in 2021 [12]. Other studies also found there are no changes in the demographics of pediatric trauma and forearm fractures pattern during the pandemic [13,14,15]. Moreover, there are reports showing an increase in the incidence of pediatric trauma during the pandemic [16,17]. These results may find some explanation in the fact that with schools closed and recreational facilities limited, children spend more time at home, often engaging in less supervised activities or play. Also, during the pandemic, there was a shift in children’s physical activities. This change in environment may have contributed to a rise in home-related injuries, including forearm fractures, as children engaged in makeshift play or risky behaviors in the absence of structured activities. Moreover, in 2021 the governmental restrictions were not as hard as in the previous pandemic year, so children had more opportunities to sustain injuries. This is consistent with several studies which demonstrated a reverse in the year 2021 of the initial decline in 2020 in the number of pediatric fractures [17,18,19].

Another important parameter assessed by this study was the potential shift during the pandemic in the anatomopathological features of the forearm fractures in children. Usually, the site of the forearm fracture, the involved bones, and the nature and degree of displacement should be in direct relations with the severity and the mechanism of the trauma sustained by the patient. Indeed, because of the restrictions during the COVID-19 pandemic, the nature of pediatric trauma changed significantly with several statistical analyses showing a significant decrease in motor and sport related trauma [13,20,21]. Accordingly, there are reports showing that the pre-pandemic anatomopathological pattern of pediatric trauma, including forearm fractures has changed [15,19,21,22]. For instance, one study from Turkey reported an increase in the distal radius fractures in children during the pandemic period [22]. In our study, we only observed an increase in the incidence of angulated fractures in the PG group. Otherwise, there were no actual shifts in the fracture patterns between NPG and PG. The site of the fractures and the bones involved were similar, as well as the number of complex or open fractures. However, not finding any significant changes in the type and site of forearm fractures was to be expected, considering that the pandemic and the restrictions had no impact on the number as well as the demographics of the patients in 2021.

When reviewing the therapeutic characteristics during the pandemic and pre-pandemic periods, we found significant differences in several aspects. First, we noticed a significant decrease in the hospitalization rates with a 52% decline in 2021. Also, there was a 60% decline in the hospitalization period (from 3.02 to 1.8 days/patient). This is consistent with every epidemiological study assessing the pandemic period [13,14,15,16,17,18,19,20,21,22,23]. During the pandemic, most hospitals adopted modified protocols to minimize exposure risk and limit the burden over the health system. In some countries, there was also an increase in the use of telemedicine with a direct impact over the number of unnecessary presentations to the Emergency Departments [19,23]. Accordingly, to a survey answered by 63 orthopedic trauma specialists from 28 different countries, 91% of participating hospitals had a significantly lower-case load during the COVID-19 pandemic [23].

There is a striking contrast between pre- and pandemic years concerning the treatment strategies of fractures in children. During the pre-pandemic years there was an increasing tendency towards a more aggressive, surgical treatment of pediatric forearm fractures [8]. After the outbreak of the COVID-19 pandemic, the tendency shifted 180^0^ towards non-surgical management, with increased reliance on splinting and casting methods to reduce the need for hospitalization and follow-up visits [15,18,19]. However, our results are in contrast with most of these studies. The pre- and pandemic treatment strategies did not differ significantly in our group of patients, with most of the cases being treated by non-surgical means. This non-shift in strategies used for forearm fractures treatment in our center can only mean that in the period preceding the pandemic, the indication for surgery was based on well set criteria and there was no tendency towards overtreatment. The increase in the use of TEN for diaphyseal fractures during pandemic is probably related to the tendency of reducing the number of postoperative visits, since this method of osteosynthesis offers better stability of the fracture site and eliminates the need for additional cast immobilization [24].

During the pandemic, there was a concern that delayed or suboptimal treatment and follow-up could lead to increased complications, such as secondary displacement, malunion or nonunion of fractures in children. Our findings indicated that the pandemic had no influence over the rate of complication. The complication rate was similar between the groups and all of them were related to secondary displacement of the fractures. There were no vascular or nerve injuries, no infections, and no compartment syndrome and all were successfully treated. Payr et al. reported a similar finding; the overall outcome of patients was not directly influenced by the reduced hospital capacity during the pandemic [25]. Follow-up of the patients it is an essential step in the treatment of forearm fractures in children. Adequate monitoring and rehabilitation can significantly reduce the risk of complications. During the pandemic, the follow up strategies were also altered to minimize the exposure risk to COVID-19. The number of followed-up patients and the number of visits per patient dropped significantly during the pandemic. This does not necessarily mean that those patients were abandoned during the pandemic. Some of them remained in contact with the family doctors and others kept in touch with the physicians by phone. Unfortunately, telemedicine was not an option in Romania at that stage. However, this decrease in the number of cases followed-up by our institution had no impact on the complication rate and the outcomes of the treatment. We have not experienced an increase in the complication rate, secondary displacement, and the need for reintervention. This is a valuable lesson that can be used further on in our treatment strategies, that hospitalization rate and the follow-up visits can be reduced with the same good results.

This study not only provides valuable insights into the impact of the COVID-19 pandemic on forearm fractures in children in Romania, but also outlines potential directions for future research. The findings contribute to the broader medical understanding of how global health crises, such as the COVID-19 pandemic, can influence pediatric trauma patterns and healthcare delivery. By exploring these shifts in injury patterns and management strategies, this research lays the groundwork for further investigations into long-term outcomes and the optimization of pediatric trauma care during emergencies.

This study has some limitations. First, in this study we only covered two years, one pre- and one during the pandemic. Even though the number of cases were sufficient for a good statistical analysis, there might be biases since, especially during the pandemic medical strategies and social limitations varied significantly in the distinct stages of the pandemic. Notably, the social limitations were significantly relieved in the year 2021 compared to the previous pandemic year. Also, we only used the database of our medical institution. Even though our institution is the sole one dealing with trauma in children in the region, there might have been cases that addressed other medical institutions. However, these potential limitations did not significantly affect the results of the study since the epidemiological features of the cases in this series are in concordance with our previous epidemiological studies [2].

## 5. Conclusions

The COVID-19 pandemic has had little impact on the epidemiology and management of forearm fractures in children. The incidence and the anatomopathological features of fractures were similar in the pandemic and non-pandemic years. The treatment options also did not vary significantly from pre- and pandemic periods. The only significant change in the medical strategy in our series was towards reducing the hospitalization rate and duration, reducing the follow-up visits. Finaly, these changes had little or no impact on the actual treatment methods and the results of the treatment.

## Figures and Tables

**Table 1:** Anatomo-pathological characteristics: site of the fracture; displacement/angulation of the bone fragments (Independent sample *t*-tests were used to compare the two groups).

		Non-Pandemic (2019)				Pandemic (2021)					
		Non-Displaced	Angulated	Displaced	2019	Non-Displaced	Angulated	Displaced	2021	Cumulated	
Radius	Proximal	23	2	11	36	16	10	8	34	70	897
	Distal	285	46	20	351	238	65	24	327	678	
	Diaphyseal	12	16	4	32	4	13	4	21	53	
	S-H	6	1	45	52	0	11	49	51	06	
Ulna	Proximal	9	0	0	9	8	0	1	9	18	49
	Distal	10	0	1	11	6	0	1	7	18	
	Diaphyseal	6	1	0	7	2	3	1	6	13	
	S-H	0	0	0	0	0	0	0	0	0	
Both bones	Proximal	3	0	2	5	5	1	2	8	13	457
	Distal	45	34	51	130	31	42	36	109	239	
	Diaphyseal	6	45	25	76	15	66	30	111	187	
	S-H	0	1	11	12	2	0	4	6	18	
Total		404	146 *	170		326	210 (*p* < 0.01) *	147			
		720				683					1403

Diaphyseal = diaphyseal fracture, Salter–Harris (S-H) = fracture that involves the growth plate. * Significantly statistical differences.

**Table 2:** Treatment options in relation to fracture site (Independent sample *t*-tests were used to compare the two groups).

		Non-Pandemic (2019)								Pandemic (2021)							
		Non-Surgical				Surgical				Non-Surgical				Surgical			
		Cast		Closed Reduction		K-Wire		TEN		Cast		Closed Reduction		K-Wire		TEN	
Proximal	Radius	24	72%	3	8%	1	2%	8	18%	21	66%	5	12%	1	6%	7	16%
	Ulna	9		0		0		0		8		0		1		0	
	Both bones	3		1		0		1		5		1		1		1	
Distal	Radius	293	72%	33	13%	27	15%	0	0.4%	251	66%	56	20%	19	11%	1	0.8%
	Ulna	10		0		0		1		6		0		1		0	
	Both bones	51		31		49		1		38		36		31		3	
Diaphyseal	Radius	14	23%	12	38%	5	19% *	2	20% *	9	26%	8	39%	0	3% *	4	32% *
	Ulna	6		1		0		0		2		3		0		1	
	Both bones	7		32		17		21		25		44		5		38	
Salter Harris	Radius	5	8%	33	61%	12	30%	1	1%	4	10%	33	72%	8	18%	0	0
	Ulna	0		0		0		0		0		0		0		0	
	Both bones	0		6		6		0		1		4		1		0	
Total		422		151		117		34		370		190		68		55	
		573				152				560				123			
		725								683							
	1408 **																

* Significantly statistical differences, ** Total number of procedures, including re-operation.

## Data Availability

The data will be available on request from the corresponding author. The data are not publicly available due to ethical restrictions.

## References

[1] Cintean R., Eickhoff A., Zieger J., Gebhard F., Schütze K. (2023). Epidemiology, patterns, and mechanisms of pediatric trauma: A review of 12,508 patients. Eur. J. Trauma Emerg Surg..

[2] Adam O., Horhat F.G., Amaricai E., David V.L., Derzsi Z., Boia E.S. (2020). Upper Extremity Fractures in Children-Comparison between Worldwide, Romanian and Western Romanian Region Incidence. Children.

[3] Kyriakides J., Peeters W., Ahluwalia A.K., Elvey M. (2022). Paediatric forearm fractures: Assessment and initial management. Br. J. Hosp. Med..

[4] Mmerem K., Beeharry M.W. (2023). Clinical and Radiological Outcomes of Paediatric Forearm Fractures of the Radius and Ulna Following Fixation by Intramedullary Nailing or Plating: A Systematic Review. Cureus.

[5] Filip R., Gheorghita Puscaselu R., Anchidin-Norocel L., Dimian M., Savage W.K. (2022). Global Challenges to Public Health Care Systems during the COVID-19 Pandemic: A Review of Pandemic Measures and Problems. J. Pers. Med..

[6] Knutsen Glette M., Ludlow K., Wiig S., Bates D.W., Austin E.E. (2023). Resilience perspective on healthcare professionals’ adaptations to changes and challenges resulting from the COVID-19 pandemic: A meta-synthesis. BMJ Open.

[7] Testa G., Sapienza M., Rabuazzo F., Culmone A., Valenti F., Vescio A., Pavone V. (2021). Comparative study between admission, orthopaedic surgery, and economic trends during Covid-19 and non-Covid-19 pandemic in an Italian tertiary hospital: A retrospective review. J. Orthop. Surg. Res..

[8] Darling J., Nowicka M., Niazi N., Pillai A. (2022). The effect of COVID-19 lockdowns on paediatric lower limb orthopaedic presentations. Arch. Orthop. Trauma Surg..

[9] Soni A., Garg S.K., Gupta R., Gupta P., Kansay R., Singhal A. (2021). Epidemiologic characteristics and pre-hospital care of traumatic injuries during the COVID-19 pandemic in an emerging and developing country: A single tertiary centre experience. J. Clin. Orthop. Trauma.

[10] Raitio A., Ahonen M., Jääskelä M., Jalkanen J., Luoto T.T., Haara M., Nietosvaara Y., Salonen A., Pakkasjärvi N., Laaksonen T. (2021). Reduced Number of Pediatric Orthopedic Trauma Requiring Operative Treatment during COVID-19 Restrictions: A Nationwide Cohort Study. Scand. J. Surg..

[11] Simon A.L., Kassab Hassan S., Julien-Marsollier F., Happiette A., Jehanno P., Delvaque J.G., Ilharreborde B. (2023). Descriptive analysis of pediatric orthopedic surgical emergencies during the COVID-19 lockdown: Single-center observational study in a pandemic red-zone area in France. Orthop. Traumatol. Surg. Res..

[12] Loder R.T., Johnson B.A. (2023). Changes in pediatric fracture patterns presenting to US emergency departments before, during, and after the COVID-19 pandemic. Heliyon.

[13] Olech J., Ciszewski M., Morasiewicz P. (2021). Epidemiology of distal radius fractures in children and adults during the COVID-19 pandemic—A two-center study. BMC Musculoskelet. Disord..

[14] Lapsa J., Bukola Badaki O., Xu A., Eaton C., Lee R.J., Ryan L. (2022). The COVID-19 Pandemic: Effects on Pediatric Fracture Patterns in the Emergency Department and Subspecialty Follow-up Care. J. Pediatr. Orthop..

[15] Memeo A., Priano D., Caldarini C., Trezza P., Laquidara M., Montanari L., Randelli P. (2024). How the pandemic spread of COVID-19 affected children’s traumatology in Italy: Changes of numbers, anatomical locations, and severity. Minerva Pediatr..

[16] Flynn-O’Brien K.T., Collings A.T., Farazi M., Fallat M.E., Minneci P.C., Speck K.E., Van Arendonk K., Deans K.J., Falcone R.A., Foley D.S. (2023). Midwest Pediatric Surgery Consortium. Pediatric injury trends and relationships with social vulnerability during the COVID-19 pandemic: A multi-institutional analysis. J. Trauma Acute Care Surg..

[17] Choi A., Bae W., Kim K., Kim S. (2021). Impact of Covid-19 on the Visit of Pediatric Patients with Injuries to the Emergency Department in Korea. Children.

[18] Zacay G., Modan-Moses D., Tripto-Shkolnik L., Levy-Shraga Y. (2022). Decreases in pediatric fractures during the COVID-19 pandemic—A nationwide epidemiological cohort study. Eur. J. Pediatr..

[19] Sugand K., Park C., Morgan C., Dyke R., Aframian A., Hulme A., Evans S., Sarraf K.M., Baker C., Bennett-Brown K. (2020). Impact of the COVID-19 pandemic on paediatric orthopaedic trauma workload in central London: A multi-centre longitudinal observational study over the “golden weeks”. Acta Orthop..

[20] Dhillon M.S., Kumar D., Saini U.C., Bhayana H., Gopinathan N.R., Aggarwal S. (2020). Changing Pattern of Orthopaedic Trauma Admissions During COVID-19 Pandemic: Experience at a Tertiary Trauma Centre in India. Indian J. Orthop..

[21] Baxter I., Hancock G., Clark M., Hampton M., Fishlock A., Widnall J., Flowers M., Evans O. (2020). Paediatric orthopaedics in lockdown: A study on the effect of the SARS-CoV-2 pandemic on acute paediatric orthopaedics and trauma. Bone Jt. Open.

[22] Oguzkaya S., Misir A., Ozcamdalli M., Eken G., Kizkapan T.B., Kurk M.B., Uzun E. (2021). Impact of the COVID-19 pandemic on orthopedic fracture characteristics in three hospitals in Turkey: A multi-center epidemiological study. Jt. Dis. Relat. Surg..

[23] Lezak B.A., Cole P.A., Schroder L.K., Cole P.A. (2020). Global experience of orthopaedic trauma surgeons facing COVID-19: A survey highlighting the global orthopaedic response. Int. Orthop..

[24] Adam O., David V.L., Horhat F.G., Boia E.S. (2020). Cost-Effectiveness of Titanium Elastic Nail (TEN) in the Treatment of Forearm Fractures in Children. Medicina (Kaunas).

[25] Payr S., Dangl T., Schuller A., Scheider P., Chocholka B., Jaindl M., Schwendenwein E., Tiefenboeck T.M. (2022). Course of Treatment and Short-Term Outcome of Surgically Treated Paediatric Upper Limb Fractures during the COVID-19 Pandemic-Experiences of a Level 1 Trauma Centre in Central Europe. Children.

